# Determinants of venom-specific IgE antibody concentration during long-term wasp venom immunotherapy

**DOI:** 10.1186/s12948-015-0036-6

**Published:** 2015-12-15

**Authors:** Valerio Pravettoni, Marta Piantanida, Laura Primavesi, Stella Forti, Elide A. Pastorello

**Affiliations:** Clinical Allergy and Immunology Unit, Foundation IRCCS Ca’ Granda, Ospedale Maggiore Policlinico, Milan, Italy; Unit of Audiology, Foundation IRCCS Ca’ Granda, Ospedale Maggiore Policlinico, Milan, Italy; Unit of Allergology and Immunology, Niguarda Ca’ Granda Hospital, Milan, Italy

**Keywords:** Hymenoptera venom allergy, Hymenoptera venom immunotherapy, Specific IgE levels, VIT long-lasting protection, VIT discontinuation

## Abstract

**Background:**

Venom immunotherapy (VIT) is an effective treatment for subjects with systemic allergic reactions (SR) to Hymenoptera stings, however there are few studies concerning the relevance of the venom specific IgE changes to decide about VIT cessation. We assessed IgE changes during a 5-year VIT, in patients stung and protected within the first 3 years (SP 0–3) or in the last 2 years (SP 3–5), and in patients not stung (NoS), to evaluate possible correlations between IgE changes and clinical protection.

**Methods:**

Yellow jacket venom (YJV)-allergic patients who completed 5 years of VIT were retrospectively evaluated. Baseline IgE levels and after the 3rd and the 5th year of VIT were determined; all patients were asked about field stings and SRs.

**Results:**

A total of 232 YJV-allergic patients were included and divided into the following groups: 84 NoS, 72 SP 0–3 and 76 SP 3–5. IgE levels decreased during VIT compared to baseline values (χ^2^ = 346.029, *p* < 0.001). Recent vespid stings accounted for significantly higher IgE levels despite clinical protection. IgE levels after 5 years of VIT correlated significantly with Mueller grade (F = 2.778, *p* = 0.012) and age (F = 6.672, *p* = 0.002). During follow-up from 1 to 10 years after VIT discontinuation, 35.2 % of the contacted patients reported at least one field sting without SR.

**Conclusions:**

The yellow jacket-VIT temporal stopping criterion of 5 years duration did not result in undetectable IgE levels, despite a long-lasting protection. A mean IgE decrease from 58 to 70 % was observed, and it was less marked in elderly patients or in subjects with higher Mueller grade SR.

## Background

Venom immunotherapy (VIT) is an effective treatment for patients suffering from hymenoptera venom allergy (HVA) with severe systemic reaction (SR) and documented sensitization to the causative venom [1].

The optimal duration of VIT necessary to achieve long-term protection has been evaluated in several studies, aimed to identify useful parameters for a safe stopping [2–4]. The initially identified criterion was the development of negative skin tests and/or serum specific IgE (sIgE) tests [5]. However, it was later noted that such outcome was rarely obtained, and that patients with positive sIgE were clinically protected from stings [2–6]. Thus, a VIT duration of at least 5 years was suggested, ideally accompanied by a decline in skin tests and sIgE levels [2–4, 7–11].

According to the latest guidelines, the decision to stop VIT must consider some risk factors for a future relapse, such as patient’s age, type of venom, severity of pre-VIT reaction, occurrence of SR during VIT, and likelihood of future stings [12, 13]. Thus, the physician may be reluctant to stop VIT even when the temporal criterion is reached, because studies evaluating the relevance of the observed declines in sIgE to decide about VIT cessation are scarce, especially regarding patients not stung during VIT, in whom the actual clinical protection is unknown.

In this study, we retrospectively evaluated the decrease in sIgE over 5 years of VIT in 3 groups of yellow jacket venom (YJV)-allergic patients: subjects stung and protected within the first 3 years or within the last 2 years of VIT, and patients not stung during the VIT course. Furthermore, we followed these three groups of patients regarding further field stings after VIT cessation, to assess the long-lasting protection of VIT and the occurrence of reactions in patients who were not stung during VIT.

## Methods

### Patients

For this retrospective study, we used our hospital database and included YJV-allergic patients who completed 5 years of VIT without SR due to venom injections at any point of VIT. All patients met the VIT admission criteria and were treated in the Clinical Allergy and Immunology Unit, Foundation IRCCS Ca’ Granda, Ospedale Maggiore Policlinico, Milan, Italy. Patients with elevated serum tryptase (>20 ng/mL) were excluded to avoid any mast cell disorder interference. Anamnesis were carefully documented, including the number of stings. SRs were classified according to Mueller grades [14].

All patients underwent VIT for at least 5 years with a maintenance dose of 100 μg of YJV (*Vespula spp.*) administered subcutaneously every 5 weeks, without changing the maintenance interval during the course of treatment. The VIT build-up phase was performed using a protocol that combines an initial rush session (first day 0.01 + 0.1 + 1 + 3 μg, cumulative dose 4.11 μg) followed by weekly injections of 10, 20, 40, 70, 100 μg [15]. At the 3rd and the 5th year of VIT, the patients underwent subsequent diagnostic tests (skin tests and sIgE measurements). The occurrence of field stings and the patient’s reaction were also documented.

The selected patients were divided into three groups: patients who were not stung (NoS) and patients who were stung and protected (SP) before the 3rd year (SP 0–3) or between the 3rd and the 5th year of VIT (SP 3–5); nobody among the SP patients experienced any SR after field stings.

### Study design

The primary aims of the study were: (1) to evaluate the mean decrease in YJV-sIgE in all the patients and in patients stung in the first 3 years or in the last 2 years of VIT, and (2) to compare the mean YJV-sIgE decrease between patients stung and protected during VIT and patients not stung during VIT.

The secondary aims were: (1) to assess possible correlations between decrease in sIgE and patients’ risk factors (age, reaction severity and number of stings), and (2) to assess the long-lasting protection in our patients by means of a phone follow-up.

### Specific IgE level measurement

YJV-sIgE levels were measured in kUA/L by means of ImmunoCAP System (Phadia, Uppsala, Sweden) according to the manufacturer’s instructions. Briefly, the allergen of interest is covalently coupled to ImmunoCAP and reacts with the sIgE in the patient sample; after washing, enzyme-labeled antibodies against IgE are added. After incubation and washing of the unbound enzyme-labeled anti-IgE, the bound complex is incubated with a developing agent, and finally the fluorescence of the eluate is measured. The higher the response value, the more sIgE is present in the serum sample. The responses are transformed into concentration by means of a calibration curve. The assay is highly automated and supplied of calibration curve and control curve, with a calibrator range 0–100 kUA/L. Clinical performance expressed as sensitivity (84–95 %) and specificity (85–94 %) and stability of the results have been reported from previous multicentric studies [16–18]. The tests were performed at baseline (CAP0) and after 3 (CAP3) and 5 (CAP5) years of VIT.

### Statistical methods

We used: ANOVA analysis for repeated measures and the Wilcoxon post hoc test to evaluate the differences between the CAP values; ANOVA univariate analyses with the Bonferroni post hoc correction to evaluate the differences in each CAP value between the three groups; multivariate analysis for repeated measures (MANOVA) to evaluate the differences in the overall CAP values between the three groups as well as the effects of age, gender, Mueller grade and number of stings; Pearson’s index to evaluate the correlations between continuous variables; the exact non-parametric Wilcoxon test to evaluate variables on small samples. A *p* value <0.05 was considered statistically significant. Data were analyzed using the SPSS^®^ program release 17.0 (SPSS Inc., Chicago, IL).

## Results

A total of 232 YJV-allergic patients (144 males, 88 females; mean age 45.05 ± 15.48 years) who completed 5 years of VIT were included in the study. Among them, 84 patients (53 males, 31 females) were never stung during VIT (group NoS), 72 patients (47 males, 25 females) were stung without SR by vespids within the first 3 years of VIT (group SP 0–3) and 76 patients (44 males, 32 females) were stung and protected during the last 2 years of VIT (group SP 3–5). No patient experienced SR during VIT as a result of field stings or as an adverse reaction to immunotherapy itself. The three groups had no statistically significant differences in mean age, number of stings before VIT or severity of sting reactions. The pre-VIT sting reactions were distributed as showed in Table 1. Considering other hypersensitivities, 22 patients had pollen allergies (11 NoS, 4 SP 0–3, 7 SP 3–5) and 17 had drug hypersensitivities (5 NoS, 9 SP 0–3, 3 SP 3–5).

**Table 1:** Patients’ Mueller grade reaction

	Mueller I	Mueller II	Mueller III	Mueller IV
NoS	9	16	33	26
SP 0–3	8	18	25	21
SP 3–5	8	21	26	21
Total	25	55	84	68

Mueller grade reaction of our total study population, divided in patients never stung (NoS), stung in the first 3 years (SP 0–3), and stung in the last 2 years (SP 3–5) of venom immunotherapy

### Evolution of IgE values during 5 years of VIT

**Fig. 1:**
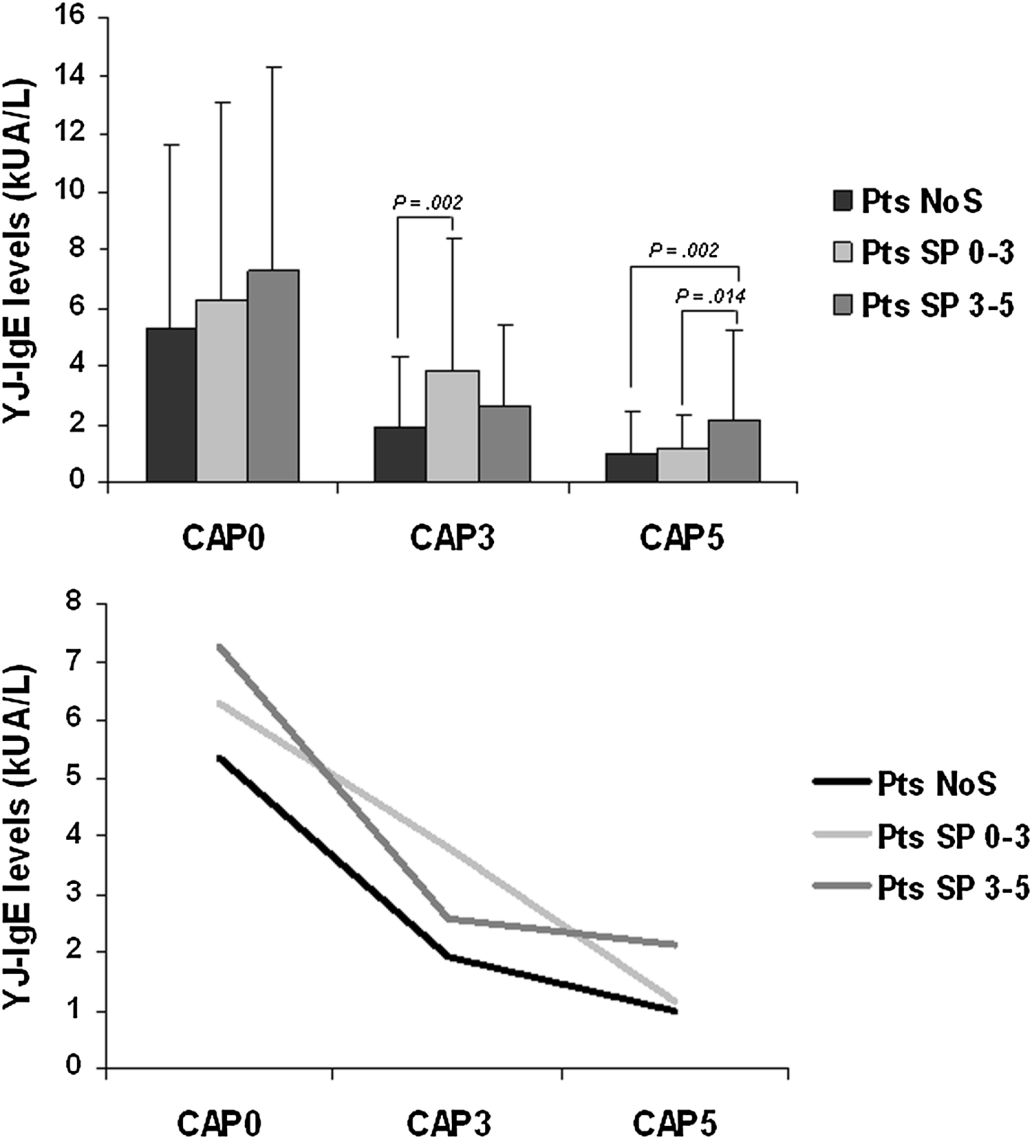
Decreases of IgE levels during VIT. Decreases in sIgE levels over 5 years of VIT in three groups of YJ-allergic patients (*NoS* not stung, *SP 0–3* stung and protected within the first 3 years of VIT, *SP 3–5* stung and protected in the last 2 years of VIT)

For the whole cohort (n = 232), YJV-sIgE levels decreased during VIT (Fig. 1) (χ^2^ = 346.029, *p* < 0.001). This finding was confirmed by post hoc tests (CAP0–CAP3: Z = −12.173, *p* < 0.001; CAP3–CAP5: Z = −11.038, *p* < 0.001; CAP0–CAP5: Z = −12.850, *p* < 0.001).

CAP5 and CAP3 were significantly different among the three groups, whereas no significant differences were found between the three groups at baseline (Table 2).

**Table 2:** Statistical results of IgE variations during VIT

YJ-IgE levels (kUA/L)	Group			ANOVA		Post-Hoc test		
						NoS vs SP 0–3	NoS vs SP 3–5	SP 0–3 vs SP 3–5
	NoS	SP 0–3	SP 3–5	F	*P*	*P*	*P*	*P*
						(95 % CI)	(95 % CI)	(95 % CI)
CAP0	5.34 ± 6.26	6.27 ± 6.81	7.27 ± 7.05	1.663	0.192	1.00 (−3.53; 1.66)	0.209 (−4.49; 0.62)	1.00 (−3.65; 1.66)
CAP3	1.91 ± 2.42	3.81 ± 4.62	2.59 ± 2.80	6.261	*0.002*	*0.002* (−3.20; −0.59)	0.616 (−1.96; 0.61)	0.085 (−0.11; 2.55)
CAP5	0.97 ± 1.44	1.15 ± 1.17	2.13 ± 3.11	6.948	*0.001*	1.00 (−0.98; 0.63)	*0.002* (−1.95; −0.36)	*0.014* (−1.81; −0.16)

Statistically significant *P* values are in italics

Mean CAP values (±standard deviation) by group (*NoS* not stung, *SP* 0–3, stung and protected within the first 3 years of VIT, *SP 3–5* stung and protected in the last 2 years of VIT) and results of statistical analysis

The overall percentage reduction in CAP at the first (3rd year) and last (5th year) control were 44.2 and 34 %, respectively; the mean CAP percentage decrease between the baseline and the 5th year control reached 65.6 %, unlike the statistical analysis performed on skin test results that did not detect any significant differences (data not shown).

Considering CAP3 values, NoS patients had YJV-sIgE levels significantly lower than SP 0–3 patients; those who had experienced a more recent sting (SP 0–3) showed significantly higher CAP values than patients who were not stung (NoS). No significant difference was found between NoS and SP 3–5 patients, and between SP 0–3 and SP 3–5 (Table 2). The patients who were not stung during the first 3 years of VIT (NoS and SP 3–5) showed similar mean percentage reductions in CAP values, 48.2 and 53.7 %, respectively, while the reduction of the SP 0–3 patients was only 30.7 % (Table 3).

**Table 3:** IgE percentage reduction during VIT related to field sting

YJ-IgE levels (kUA/L)	Group	Mean ± SD	Min	Max	Mean percentage reduction (%)
CAP0–CAP3	NoS	3.81 ± 5.13	0.01	27.59	48.2
	SP 0–3	2.61 ± 3.53	0.00	21.22	30.7
	SP 3–5	4.75 ± 5.32	0.00	25.07	53.7
CAP3–CAP5	NoS	0.97 ± 1.60	0.00	11.97	37.1
	SP 0–3	2.68 ± 4.13	0.00	20.97	50.3
	SP 3–5	0.82 ± 1.03	0.00	6.40	17.0
CAP0–CAP5	NoS	4.39 ± 5.48	0.00	27.54	68.6
	SP 0–3	5.15 ± 6.16	0.00	30.87	70.5
	SP 3–5	5.24 ± 5.55	0.12	25.30	57.7

Absolute (mean ± standard deviation SD, minimum and maximum) and percentage reductions of CAP values by group (*NoS* not stung, *SP 0–3* stung and protected within the first 3 years of VIT, *SP 3–5* stung and protected in the last 2 years of VIT)

At the final VIT control (CAP5), the patients who were recently stung (SP 3–5) had significantly higher CAP values than those who were not stung or were stung during the first 3 years of VIT (Table 2). The sIgE final percentage reduction in the SP 3–5 group was less than 60 % when compared to baseline, while NoS and SP 0–3 patients presented a reduction of approximately 70 % (Table 3).

### Influence of risk factors on the evolution of sIgE values

MANOVA analysis revealed that the CAP values were different during VIT (F = 28.872, *p* < 0.001) in the different groups (NoS, SP 0–3, SP 3–5). CAP values were also significantly related to Mueller grade (F = 2.778, *p* = 0.012) and age (F = 6.672, *p* = 0.002): a higher Mueller grade and a more advanced age were associated with higher CAP5 values. The Pearson’s index confirmed a significant correlation between age and CAP 0–5 reduction (r = −0.609, *p* < 0.001). The other analyzed variables (gender and number of stings) were not statistically significant.

### Follow-up after VIT discontinuation

All the patients were contacted by phone to determine if they had been stung after VIT cessation; results are reported in Table 4. We successfully contacted 159/232 (68.5 %) patients. Among the 84 NoS patients, we successfully contacted 70 (83 %) patients, 13 of which had been field-stung. 21 of the SP 0–3 patients and 22 of the SP 3–5 patients had been field-stung. A total of 56 (35.2 %) patients reported at least one field sting without any systemic reaction, from 1 to 10 years after VIT cessation. Some patients received multiple vespid stings from several months to several years after VIT cessation and did not experience any reactions.

**Table 4:** VIT discontinuation: restung patients’ follow-up

Patients	NoS during VIT	SP during VIT
Total	84	148
Recalled	70	89
SP after VIT stopping	13/70	43/89
Within 1 year after	2	16^a^
After 2–3 years	–	11^a^
After 4–5 years	6	5^a^
After 7–8 years	3^a^	7 (4; 3^a^)
After 9–10 years	2	4 (1; 3^a^)

Follow-up after VIT discontinuation: number of patients stung and protected (SP) after stopping by group and period of time

^a^At least one patient received other field stings before or after

**Fig. 2:**
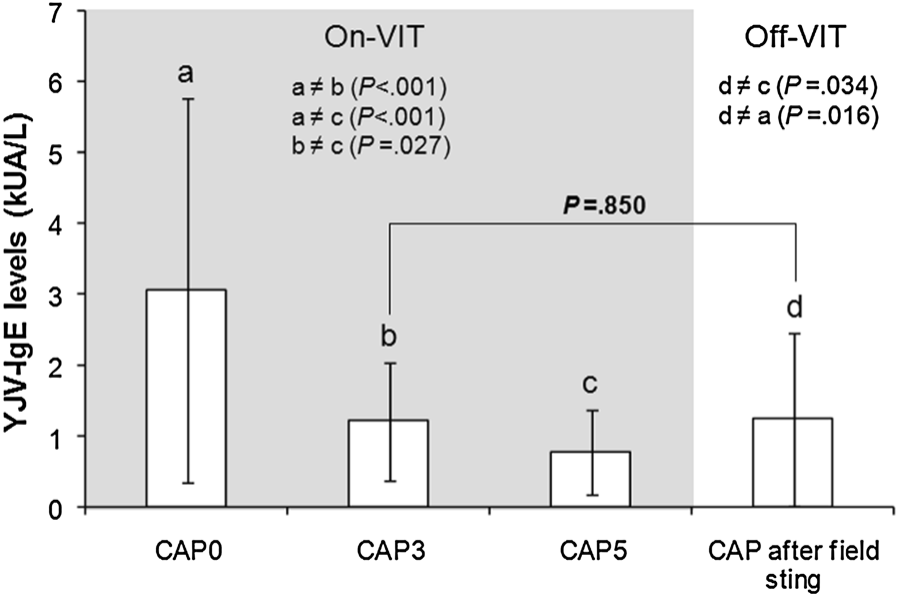
IgE levels after field sting and VIT discontinuation. Mean IgE levels in 13 patients at a field sting after VIT stopping related to their mean IgE levels during VIT (*grey part*). After field sting IgE levels increased and returned to the levels of the intermediate control (CAP3), independently from the period of field sting during VIT and from the time between VIT stopping and field sting

Despite the clinicians recommendations to come back to our Centre for sIgE level determination in case of field sting after VIT discontinuation, only 13/56 (23.2 %) patients did. These patients (eight males, five females; mean age 43.54 ± 17.55) had a well tolerated field sting from few months to 4 years after VIT stopping. All of them had already been field stung during VIT, as seven patients belonged to SP 0–3 group and six to SP 3–5 group. As shown in Fig. 2, the mean IgE level determined about 2 months after the field sting resulted significantly different from the IgE level at baseline (Z = 2.342; *p* = 0.016) and at 5th year control (Z = −2.118; *p* = 0.034), while it did not differ from 3rd year control (Z = −0.235; *p* = 0.850).

## Discussion

In this retrospective study, we selected 232 YJV-allergic patients. Serum sIgE levels determined before starting VIT, and at 3rd and 5th year controls, showed a significant decrease over time, which was an expected result. In fact, one of the immunological effects of VIT is the progressive reduction of sIgE levels [6, 19–21]. The reduction of venom sIgE during VIT is a well known effect since 1983, when the loss of venom sensitivity due to VIT was described, with IgE levels showing an initial increase, and then followed by a reduction over the 3 years of VIT [6, 22]. The sIgE reduction over the course of VIT was confirmed by many studies, performed with both children and adults: after an initial increase at maintenance dose, venom sIgE fall after 3–5 years of venom immunotherapy [19–24]. Some Authors found a small decline in the mean venom sIgE levels also during the first year after VIT was stopped, suggesting both the duration of 5-year VIT and the passage of time can play a role in the decrease of sIgE [19]. The mechanism of specific IgE reduction during immunotherapy is likely due to the cytokine shift from a T-helper 2 to a T-helper 1 dominant pattern [25, 26]. Changes in cytokine production (decrease of IL-4, increase of IFN-gamma and IL-10) has been demonstrated for VIT, also during the early phase, with potential down regulation of mast cell and basophil reactivity, and rapid desensitization in rush VIT; in the longer term, the immunological shift would result in an isotype switch from IgE to IgG [27–29].

Studies on natural history of insect sting allergy showed that, among patients with SRs and positive skin tests not treated with VIT, about 60 % had clinical re-sting reactions, with an higher rate of reactions in patients with more severe initial reactions [30]. Golden et al. [31] evaluated the changes in diagnostic tests and the risk of sting reactions in patients not admitted to VIT, demonstrating a 10–12 % per year loss in skin test positivity, with a negativization in 45 % of subjects after 4 years. Sensitization to venom may disappear in 30–50 % of cases after 5–10 years, but can also persist for many years even without sting exposure, with a 20 % chance of systemic reaction after 15 years in subject not treated with VIT. The risk of future systemic reactions depends on the severity of previous reactions: it is higher (60–70 %) in adults with severe anaphylaxis than in those with moderate (40 %) or mild (20 %) anaphylaxis [32]. In these studies, the reactivity of patients was assessed by spontaneous field stings or by deliberate sting challenges. Each method has its limitations: for field sting there is the uncertainty of the insect identification, for sting challenge (at least concerning vespids) there is the uncertainty of the amount of venom [33]. Indeed, there are studies demonstrating that a single negative sting challenge is not decisive to predict patient protection [33–35]. Up to now, in many European Countries, including Italy, the sting challenge is not recommended because is considered poorly reproducible and hazardous [12]. In one of the first surveys on VIT [6], 71 % of patients were clinically protected after a field sting, even though only a minority of them had negative sIgE after 3 years of VIT. Golden found a similar low percentage (approximately 30 %) of skin test negativization after 5 years of VIT, though all patients were sting challenge negative [36]. In the present study, at VIT cessation none of our patients exhibited a complete negativization of YJV-sIgE, even though the majority of them (63.8 %) had at least one well tolerated vespid field sting. Hence, a correlation between clinical protection and negativity of sIgE may not exist and should not be considered a reliable indicator of successful VIT.

To our knowledge, this is the first study investigating the associations of the risk factors for relapse after VIT with the evolution of sIgE levels during VIT. In our elderly patients and in patients with higher Mueller grade reactions, we observed a smaller decrease in sIgE during the VIT course; our results correlate well with the previously published data [7, 8, 11, 15]. A clinician evaluating the decision to stop VIT could take into consideration that patients with higher Mueller grade reactions and advanced age could present a smaller decrease of venom-sIgE, without necessarily invalidating the efficacy of VIT.

Analyzing the sIgE levels over time and the differences between the three groups (NoS, SP 0–3 and SP 3–5), we found a significant difference in CAP values at the 3rd year control between NoS and SP 0–3 patients, because of the sIgE increase associated to the recent vespid sting in the latter group. There was no significant difference between SP 0–3 and SP 3–5 patients, though the first group experienced a recent vespid sting, probably because of the higher CAP0 value presented by SP 3–5 patients. NoS and SP 3–5 patients exhibited a mean percentage reduction between CAP0 and CAP3 values of approximately 50 %, while SP 0–3 patients exhibited a less marked decrease (30 %).

At the 5th year control SP 0–3 patients had reduced CAP values, that were comparable with those of NoS patients. There was a significant difference between NoS, SP 0–3 and SP 3–5 patients. Considering the mean percentage reduction between CAP3 and CAP5, SP 3–5 patients achieved only a 17 % reduction, while NoS and SP 0–3 patients experienced reductions of 37 and 50 %, respectively. Therefore, subjects who experienced a recent vespid sting, even if clinically protected, had significantly higher CAP values than patients who were not recently stung. Hence, higher than expected mean CAP values after 5 years of treatment in patients with recent field stings should not be considered as a criterion for VIT continuation. Despite sIgE levels has been evaluated after some years, we consider our CAP results to be reliable, as the stability, the reproducibility and the high degree of standardization of the ImmunoCAP assay have been previously demonstrated, retesting the same serum sample after storage at −20 °C over an 8-year period and confirming the reproducibility of the quantitative measurements of sIgE [18]. Furthermore, the coefficient of variation of the assay is very low (≤10 %) and independent of allergen specificity and IgE levels [37]. In our study the overall variability of sIgE detection at 3rd and 5th year of VIT is higher than 10 % of the coefficient of variation, so we can state that a real decrease in sIgE detection occurred.

After VIT stopping, 13 patients (seven belonging to SP 0–3 group and six to SP 3–5 group) underwent laboratory analyses after field stings. In this few patients sIgE levels increased after the field sting, resulting similar to the 3rd year control (Fig. 2).

We also performed phone interviews, asking patients if they had been stung after VIT stopping, to evaluate the protection rate. Among the 159 responders (70 NoS and 85 SP), 56 (35.2 %) reported one or more well-tolerated stings; almost all patients were clinically protected until 3 years after VIT cessation, and some were protected up to 10 years post-VIT. Only 19 % of NoS patients were stung after VIT termination; SP patients received more field stings than NoS patients after stopping VIT, most likely because they were less fearful to expose to risky outdoors situations. The follow-up survey after VIT cessation determined that all recalled patients, after 5 years VIT, were clinically protected for up to 10 years.

Considering the percentage reductions after 5 years of VIT, we observed that patients stung or not stung during the first 3 years showed a mean CAP reductions of roughly 70 % compared to their baseline values. For patients who were stung within the last 2 years of VIT, the mean CAP value decreased by roughly 58 % compared to baseline.

## Conclusions

In conclusion, when a patient fulfills the temporal criterion for VIT duration (at least 5 years) but still has positive sIgE tests, a mean IgE decrease ranging from 58 to 70 % compared to baseline is likely to be expected. This decrease could be less striking in elderly patients or in subjects with a higher pre-treatment Mueller grade SR. Anyhow, the measurement of venom-specific IgE levels remains the best in vitro parameter to monitor VIT, as demonstrated by follow-up studies of patients with long-lasting protection.

## Abbreviations

VIT: venom immunotherapy
SR: systemic reaction
YJV: yellow-jacket venom
YJ: yellow-jacket
sIgE: serum specific IgE
SP 0–3: patients stung and protected during the first 3 years of VIT
SP 3–5: patients stung and protected within the last 2 years of VIT
NoS: patients not stung during VIT
CAP: IgE levels detection by ImmunoCAP System
ANOVA: analysis of variance
MANOVA: multivariate analysis of variance
